# Associations between Intra-Individual Variability of Reaction Time and Cognitive Function in Cognitively Normal Senior Adults: Still beyond Good or Bad?

**DOI:** 10.3390/geriatrics1020013

**Published:** 2016-06-03

**Authors:** Hanna Lu, Sandra S. M. Chan, Linda C. W. Lam

**Affiliations:** 1 Department of Psychiatry, The Chinese University of Hong Kong, G/F Multicenter, Tai Po Hospital 100000, Hong Kong, China; schan@cuhk.edu.hk (S.S.M.C.); cwlam@cuhk.edu.hk (L.C.W.L.); 2 Department of Psychiatry, Guangzhou Brain Hospital, The Affiliated Brain Hospital of Guangzhou Medical University, Guangzhou 510370, China

**Keywords:** intra-individual variability, short-term fluctuations, MoCA, reaction time, cognitive ageing, age-related cognitive decline

## Abstract

***Background:*** Intra-individual (IIV) of reaction time (RT), as the short-term fluctuations of RT-based performance, has been reported to be susceptible to cognitive ageing. The current study aimed to examine IIV of RT and its relationships with cognitive performance in cognitively normal senior adults. ***Methods:*** We evaluated 137 community-dwelling elderly (mean age: 72.41 ± 3.99) with a comprehensive neuropsychological battery and a RT-based test. Intraindividual coefficient of variation of reaction time (ICV-RT) was used to evaluate the IIV. Pearson’s correlation and hierarchical multiple regression analyses were employed to assess the relationships between IIV and the scores of cognitive function. ***Results:*** Advancing age was accompanied with declined cognitive function and increased IIV. ICV-RT was negatively correlated with the score of Montreal Cognitive Assessment Hong Kong version (HK MoCA) across three types of flanker. Hierarchical multiple regression showed that ICV-RT was a significant predictor of HK MoCA (β = −0.294, *p* = 0.001). ***Conclusion:*** Increased IIV is consistently associated with and contributed to the age-related decline of cognitive performance in senior adults. The utility of IIV in predicting further deterioration should be carefully postulated with prospective studies.

## 1. Introduction

Intra-individual variability (IIV), as a facet of within-person variation, has been regarded as a measure of short-term fluctuations in an individual’s performance [1]. Throughout the adulthood, IIV represents a dynamic behavioral characteristic that appears to progressively change with age. An emerging body of evidence has demonstrated a dynamic pattern of IIV across life span, such as IIV is greater in childhood, slightly decreased in adolescence and early stage of adulthood, then keeps steady through adulthood, and with a return of rapid increase in late adulthood [2,3]. This “U-shape” pattern of IIV across life span has been considered as an adaptive behavior and illustrated as a potential role to discriminate the individuals with different age range [4]. It is not surprising that cross-sectional studies have addressed a global view of “age-IIV” correlates. While, it should be noticed that this coupling changes could be affected by cognitive status as well. For instance, compared with young adults, IIV of reaction time (RT) has been prominently increased in adults with an age of 50 and above on simple RT test, even greater on choice RT test [5,6,7]. Beyond cognitive ageing, the discriminative value of IIV has also been considered as an indicator of predicting conversion from healthy ageing to mild cognitive impairment (MCI) [8,9].

Therefore, it appears that the IIV of RT not just varies among the individuals with different age range, but also differs among the ones with different cognitive status. This phenomenon triggers another concern is whether the coupling change between age and IIV has a general effect on cognitive function or only linked to a specific cognitive domain? Besides, given the linkage between age and IIV reported in our recent work [10], we would proceed the further explorations focusing on three questions in current study: (1) Whether chronological age is associated with IIV of RT under the conditions with different cognitive demand; (2) Whether IIV of RT is associated with cognitive performance and to what extent; (3) Does IIV of RT predict the age-related change of cognitive function? Based on previous evidence, we hypothesized that short-term fluctuations on RT-based test would be increased with advancing age. In addition, we also expected IIV of RT would be associated with the scores of cognitive function and contributed to the age-related cognitive decline.

## 2. Methods

### 2.1. Participants

One hundred and thirty-seven community-dwelling Chinese adults aged from 65–80 years were recruited from another cohort study that aims to establish a detailed characterization of cognitive and healthy profiles of Chinese senior adults. The eligible participants were scheduled for a 1.5-hour in-person interview at Chen Wai Wai Vivien foundation therapeutic physical mental exercise centre. During the on-site interview, the participants received a comprehensive evaluation [10], including cognitive function, medical history, and everyday activity.

The inclusion criteria of cognitively normal elderly were as follows: (1) aged 65 years and older; (2) no significant cognitive impairment: the ones with cognitive performance within 1.5 standard deviation (SD) of the age-normal reference (see Table 1) derived from the cohort study, which presents with Clinical dementia rating scale (CDR) score equal to 0 and Cantonese version of Mini Mental State Examination (CMMSE) score of larger than 28; (3) Exclusion criteria were as follows: (1) mild or major neurocognitive disorders as defined by DSM-5 (APA, 2013); (2) presence with mood disorder, sleep disorder, and psychiatric disorders.

### 2.2. Clinical Evaluation and Cognitive Assessment

A structured neuropsychological battery was used to assess the global cognition and major domains of cognition [10]. CDR, Alzheimer’s Disease Assessment Scale (ADAS), CMMSE, and Montreal Cognitive Assessment Hong Kong version (HK MoCA) were conducted as a global measure of cognition. Three major domains of cognitive function were including [11]: (1) Short-term memory: delayed recall of words and digit span backward (DSB); (2) Attention: trail making test A (TMT-A) and digit span forward (DSF); (3) Executive function: trail making test B (TMT-B).

Cerebrovascular risks were evaluated by cumulative illness rating scale (CIRS) [12] with presence of hypertension, atrial fibrillation, heart diseases, anaemia, diabetes mellitus, and hyperlipidaemia. Cornell scale for depression in dementia (CSDD) [13], Pittsburgh sleep quality index (PSQI) [14], and activity of daily living scale (ADL) [15] were used to assess depression symptom, sleep disorder, and everyday functioning separately.

All the assessments were conducted with Chinese instructions. Ethical approval was obtained from the Joint Chinese University of Hong Kong-New territories East Cluster Clinical Research Ethics Committee. Written informed consent from all participants was obtained before the assessments.

### 2.3. Measure of Intra-Individual Variability (IIV)

**Figure 1:**
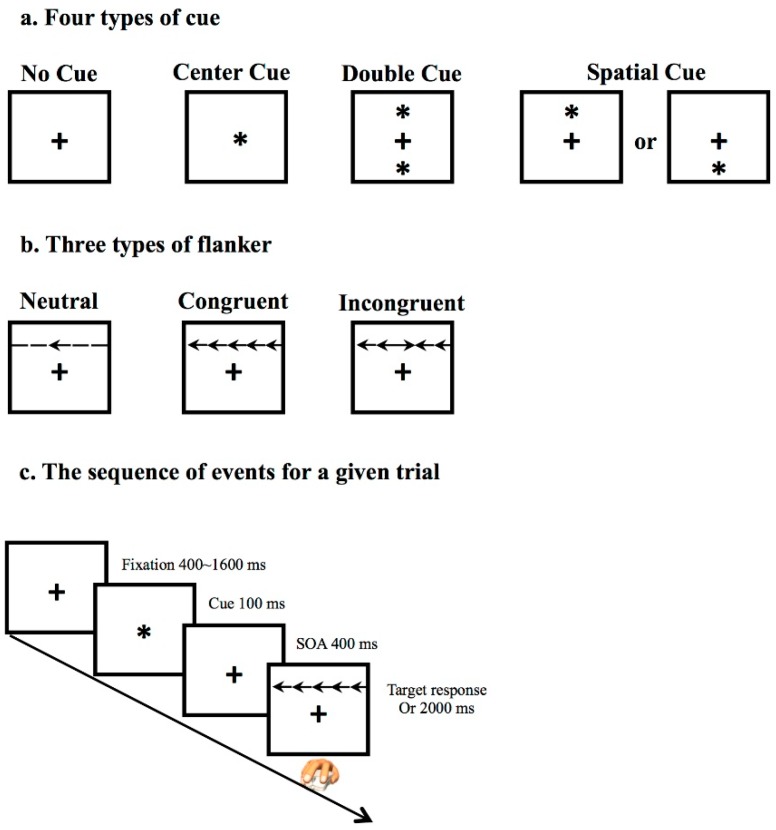
Attention network test (ANT). A schematic of the four types of cue (**a**) and three types of flanker (**b**) that are used in ANT with the time courses of events (**c**).

As described above, IIV represents the inconsistency of cognitive performance through repeated measurement. Attention network test (ANT) [16], as a well-accepted computerized test, includes 288 trials (Figure 1c) for collecting response speed, which is suitable for measuring IIV. Within the ANT paradigm, three types of flanker represent the different cognitive demand, which are: neutral, congruent, and incongruent (Figure 1b). Two indices are conduced to evaluate the ANT performance: (1) Accuracy is the degree of correctness of making a decision; (2) Reaction time (RT), as the completion time in millisecond (ms) for a given trial, is used for assessing the average processing speed and IIV of RT.

In consideration of adjusting slowing processing speed in old age, intraindividual coefficient of variation of reaction time (ICV-RT) [17,18] is used to evaluate the IIV enumerated with the formula: ICV-RT = (standard deviation of processing speed/mean of processing speed) × 100 [19]. Therefore, ICVRT_Neutral, ICVRT_Congruent, and ICVRT_Incongruent are used to describe the IIV under the conditions with different types of flanker. Higher ICV-RT indicates greater short-term fluctuations across the ANT trials.

### 2.4. Statistical Analyses

As suggested by the developer of ANT [16], median values of RT were used as raw scores to calculate the processing speed and ICV-RT. Pearson’s correlation between age, ICV-RT, and the scores of cognitive function. For significant correlations, hierarchical multiple regression analysis was employed to assess the ability of ICV-RT to predict the scores of cognitive function, after controlling for the same potential confounding factors. In the first step, age was included because of its significant relationship to cognitive function. Secondly, ICV-RT was included to predict the age-related change of cognitive performance. The Δ*R*^2^ value represented the increase in proportion of variance explained in step 1. Alpha was set as *p* < 0.05. The scores of accuracy and RT were calculated by E-Data Aid embedded in E-Prime 2.0 (Psychology Software Tools, Pittsburgh, PA, USA). The calculation of ICV-RT, Pearson’s correlation analysis and hierarchical multiple regression analysis was performed by IBM SPSS 20.

## 3. Results

### 3.1. Associations between Age and Cognitive Function

With gender and education as covariates, significant correlations were found between age and cognitive function (CDR: *r* = 0.17, *p* = 0.042; TMT-B: *r* = −0.265, *p* = 0.002). As to IIV, a significant correlation was found between age and the average ICV-RT *(r* = 0.198, *p* = 0.021), indicating that adults with older age present greater short-term fluctuations on RT-based test. Moreover, prominent association was found between age and the ICV-RT under neutral flanker (*r* = 0.259, *p* = 0.003) instead of the difficult ones (ICVRT_Congruent: *r* = 0.156, *p* = 0.072; ICVRT_Incongruent: *r* = 0.164, *p* = 0.058).

### 3.2. Associations between Age, ICV-RT, and Cognitive Function

Using age, gender and education as covariates, a significant negative relationship was found between ICV-RT and HK MoCA (Table 2). Besides, there was a trend that the associations with presence of the absolute values of correlation coefficients (*r*) became stronger when the types of flanker from lower cognitive demand (Neutral: *r* = −0.216, *p* = 0.012) to higher cognitive demand (Congruent: *r* = −0.245, *p* = 0.004; Incongruent: *r* = −0.256, *p* = 0.003). To further probe the associations between ICV-RT and cognitive function, hierarchical multiple regression analyses showed that the Δ*R^2^* change of average ICV-RT was significant (*β* = −0.294, *t* = −3.538, *p* = 0.001) when regressing out the effect of age. In addition, Δ*R^2^* changes of the ICV-RT under three types of flanker were also significant (Neutral: *β* = −0.313, *t* = −3.778, *p* < 0.001; Congruent: *β* = −0.343, *t* = −4.245, *p* < 0.001; Incongruent: *β* = −0.315, *t* = −3.859, *p* < 0.001), which indicates that increased short-term fluctuations contribute to the age-related decline of HK MoCA score.

## 4. Discussion

The present study aimed to examine the relationships between intra-individual variability of RT and cognitive performance in community-dwelling cognitively normal senior adults. As expected, we observed several prominent associations between age, ICV-RT, and scores of cognitive function. In particular, advancing age was positively correlated with ICV-RT under the condition with lower cognitive demand (*i.e.,* sample RT test with neutral flanker). In contrast, cognitive performance, such as HK MoCA, was negatively associated with the ICV-RT across different levels of cognitive demand. These findings suggest that the increased short-term fluctuations on RT-based performance might be a fundamental feature of cognitive ageing.

### 4.1. Age, IIV, and Cognitive Function

In recent years, variability has been considered as an important individual difference measure relevant to understanding age differences in brain function [20]. It is not surprising that significant elevations in IIV have been found in adults with neurological disease and dementia [21,22]. Of note, aligned with previous findings [6,9,23,24], the increased IIV across ANT trials not just correlated with age, but also reflected the age-related cognitive decline (ARCD). Given the key role that IIV plays in understanding ARCD [7,25], the consistent association between ICV-RT and HK MoCA provides an opportunity to rethink that aforementioned concern that cognitive function maybe affected by IIV as well. Indeed, further hierarchical multiple regression analysis confirmed that ICV-RT significantly contributed to the age-related decline of global cognition measured by HK MoCA.

### 4.2. IIV and Cognitive Function

The observed association between trial-to trial fluctuations on ANT and HK MoCA in cognitively normal senior adults across three types of flanker is an intriguing finding. In view of the claim that IIV with a measurement scale of millisecond (ms) in RT-based test is likely be related to cognitive function [26], the absence of correlation between TMT (with a measurement scale of second) and HK MoCA, and a linkage of IIV and HK MoCA in this study might be attributed to a more precise measurement scale. 

However, as an unexpected result, there was a trend that the correlations between IIV and HK MoCA became stronger when the ICV-RT under the conditions with lower cognitive demand (neutral) to higher cognitive demand (congruent and incongruent). Although the corresponding change of absolute value of *r* seems spurious, further hierarchical multiple regression analyses partially reproduced the above trend with increased contributions (from neutral to incongruent) to ARCD. One possible explanation would be the implicit and explicit changes inside the ageing brain. Indeed, age-related changes of cognitive function occur gradually with the reduction of dopamine D_2_ receptor in the frontal lobe from the late 20s [27], but become more noticeable in the population under cognitive ageing [28]. Evidence form positron emission tomography (PET) studies have also confirmed that the increased IIV was highly associated with the dysregulation of dopamine embedded in frontal lobe circuitry [4]. The involvement of frontal lobe circuitry might support an integrative view that more cognitive efforts are given to facilitate and maintain the consistent performance on RT-based test during the ageing process [29,30]. From this perspective, it is reasonable to postulate that the characteristics of IIV might reflect the early sign of brain dysfunction [31,32].

In the light of the preceding discussion, our results are aligned with the emerging evidence that cognitive ageing is accompanied by the dynamic changes, including increased IIV, slowing processing speed, and declined executive function [33]. At this point, we summarize two major thrusts of this study: (1) Increased IIV is associated with declined global cognition in the context of cognitive ageing; (2) IIV under different levels of cognitive demand consistently contributes to the age-related cognitive decline.

## 5. Limitations

Despite us providing an example of linking IIV to cognitive function, the results of the current study should be interpreted within its limitations. First, the classifications of cognitively normal senior adults were based on the scores of neurocognitive tests, not ascertained by neuroimaging and blood biomarkers. Second, this cross-sectional evaluation had limited power to infer any causative influence of findings. Third, all participants were from the Chinese community, which may not give an accurate reflection of the features of adults with various cultural backgrounds.

## Figures and Tables

**Table 1:** Demographics and cognitive function of healthy senior adults.

	Mean ± SD	Age-Normal Reference
Demographics		
Age	71.45 ± 3.99	70.14 ± 4.08
Gender (F/M)	61/76	105/164
Education (years)	9.30 ± 4.26	10.30 ± 4.36
CSDD	0.49 ± 1.81	0.49 ± 1.72
PSQI	5.75 ± 3.15	5.77 ± 3.53
ADL	0.99 ± 0.02	0.99 ± 0.02
Cognitive function		
CDR-SOB	0.34 ± 0.47	0.16 ± 0.30
CMMSE	28.63 ± 1.17	28.82 ± 1.05
HK MoCA	27.23 ± 1.85	27.93 ± 1.52
ADAS	4.91 ± 2.08	4.82 ± 2.24
Delayed recall	7.64 ± 1.45	7.92 ± 1.45
Trail making test A (ms)	12.90 ± 6.37	12.53 ± 6.33
Trail making test B (ms)	67.07 ± 41.26	61.23 ± 39.67
Digit span forward (DSF)	7.62 ± 1.13	7.78 ± 1.06
Digit span Backward (DSB)	3.75 ± 1.32	4.03 ± 1.38

Note: CSDD = The Cornell Scale for Depression in Dementia; PSQI = Pittsburgh Sleep Quality Index; CDR-SOB = Clinical dementia rating-sum of box; ADL = Activity of daily living scale.

**Table 2:** Associations between IIV of RT and cognitive performance.

	ICVRT-Neutral			ICVRT-Congruent			ICVRT-Incongruent	
	*r*	*p*		*r*	*p*		*r*	*p*
CDR-SOB	−0.019	0.828		0.014	0.871		−0.016	0.858
CMMSE	0.082	0.346		−0.012	0.894		−0.030	0.735
**HK MoCA**	**−0.216**	**0.012**		**−0.245**	**0.004**		**−0.256**	**0.003**
ADAS−Cog	0.017	0.844		0.022	0.798		0.108	0.217
Delayed recall	0.016	0.856		−0.014	0.876		−0.042	0.628
DSF	−0.047	0.593		−0.082	0.346		0.003	0.975
DSB	−0.047	0.593		−0.042	0.633		−0.096	0.273
TMT-A	0.094	0.284		0.115	0.188		0.027	0.755
TMT-B	0.099	0.255		0.102	0.242		0.087	0.318
